# Postural Change of the Annual Cicada (*Tibicen linnei*) Helps Facilitate Backward Flight

**DOI:** 10.3390/biomimetics9040233

**Published:** 2024-04-14

**Authors:** Ayodeji T. Bode-Oke, Alec Menzer, Haibo Dong

**Affiliations:** Department of Mechanical and Aerospace Engineering, University of Virginia, Charlottesville, VA 22903, USA; atb5dc@virginia.edu (A.T.B.-O.); dfg5nb@virginia.edu (A.M.)

**Keywords:** cicada, backward flight, body kinematics, aerodynamic force control, insect maneuverability

## Abstract

Cicadas are heavy fliers well known for their life cycles and sound production; however, their flight capabilities have not been extensively investigated. Here, we show for the first time that cicadas appropriate backward flight for additional maneuverability. We studied this flight mode using computational fluid dynamics (CFD) simulations based on three-dimensional reconstructions of high-speed videos captured in a laboratory. Backward flight was characterized by steep body angles, high angles of attack, and high wing upstroke velocities. Wing motion occurred in an inclined stroke plane that was fixed relative to the body. Likewise, the directions of the half-stroke-averaged aerodynamic forces relative to the body (local frame) were constrained in a narrow range (<20°). Despite the drastic difference of approximately 90° in body posture between backward and forward flight in the global frame, the aerodynamic forces in both flight scenarios were maintained in a similar direction relative to the body. The forces relative to the body were also oriented in a similar direction when observed during climbs and turns, although the body orientation and motions were different. Hence, the steep posture appropriated during backward flight was primarily utilized for reorienting both the stroke plane and aerodynamic force in the global frame. A consequence of this reorientation was the reversal of aerodynamic functions of the half strokes in backward flight when compared to forward flight. The downstroke generated propulsive forces, while the upstroke generated vertical forces. For weight support, the upstroke, which typically generates lesser forces in forward flight, is aerodynamically active in backward flight. A leading-edge vortex (LEV) was observed on the forewings during both half strokes. The LEV’s effect, together with the high upstroke velocity, increased the upstroke’s force contribution from 10% of the net forces in forward flight to 50% in backward flight. The findings presented in this study have relevance to the design of micro-aerial vehicles (MAVs), as backward flight is an important characteristic for MAV maneuverability or for taking off from vertical surfaces.

## 1. Introduction

Over millions of years, insects have developed techniques to generate forces and enhance their maneuverability in flight. A combination of a robust neuro-sensory system, kinematics control, and the use of different aerodynamic mechanisms makes their flight possible [1,2]. While previous works have focused on hovering and forward flight to understand the aerodynamics and mechanics of insect flight, some insects extend their flight envelope to include backward or reverse flight [3,4,5,6,7]. Examples include hovering specialists and pollinators, as well as hematophagous insects [7]. For maneuverability and other biological purposes, backward locomotion is also expressed widely in nature among fish, birds, humans, ants, etc. [8,9,10,11,12]. In insect flight particularly, backward locomotion is appropriated for prey capture, flight initiation from vertical surfaces, and obstacle avoidance; it may render immediate turning after an activity such as hematophagy or pollination unnecessary [3,6,10,13], and further investigations in this area may provide insights for and inspire micro-aerial vehicle (MAV) designs. As of yet, most high-detail studies of cicada flight aerodynamics have focused on the forward flight mode.

Vis à vis aerodynamics studies on the forward flight of cicadas have identified body-generated vortices and quantified the interaction of the wings with these body-generated vortices [14,15]. Lift enhancement on the body occurred due to wing–body interactions (WBIs), while body lift was negligible when isolated from the wings. The overall lift enhancement due to WBIs was about 19%. Despite the lift increase due to WBIs, the wings remained the dominant source of force generation [15]. By controlling the wing kinematics asymmetrically on a half-stroke basis, typical of insects that flap in an inclined stroke plane, cicadas produce 80–90% of the net aerodynamic forces during the downstroke (dorsoventral stroke), while the upstroke (ventrodorsal stroke) plays an auxiliary role, contributing only 10–20% of the net forces in forward flight [14,15]. The downstroke (DS) forces provide weight support, similar to other insects in forward flight, due to the combination of higher effective wing velocities (body + wing velocity) and angles of attack (AoAs) in comparison to the upstroke (US) [7,16]. The US is aerodynamically inactive with regard to weight support, but provides some thrust in forward flight [15]. The smaller forces produced in the US are generally due to both lower effective AoAs and wing velocities [17,18]. Additionally, prior works on cicadas have often focused on other aspects such as sound production [19], life cycle [20], energetics and thermoregulation [21], and wing surface topography [22]. The understanding of the cicada’s backward flight mode, which is an alternative to forward flight as an extra avenue for maneuverability, is incomplete compared to other aspects of its biological functions and locomotion.

Before now, insect backward flight has only been quantitatively documented in a few studies. Hawkmoths [23], butterflies [24], and dragonflies [25,26] have been shown to perform backward flight. Numerical simulations yielded detailed aerodynamic measurements that indicated that butterflies employ a body re-orientation strategy in backward flight that reverses the US and DS roles in force production compared to forward flight: in backward flight, the US provided weight support and the DS provided horizontal forces [24]. A similar force generation role reversal between forward and backward flight was also recorded for dragonflies [25]. Odonates and Dipterans have typically served as candidates for studying complex flight behaviors [2,25]; however, in this study, we investigated the backward free flight of the annual cicada, which, like other Homopterans, is not characteristically associated with excellence in maneuverability [27]. Nevertheless, after their emergence from the developmental stages underground, maneuverability is essential to cicadas’ survival in the aerial world above. To escape from predators, to navigate their arboreal environment, and for food, cicadas perform controlled maneuvers. These include forward flight, takeoff, and more exotic maneuvers like banked turns and Immelmann turns, as well as backward flight, which hitherto is an undocumented flight mode of this animal [28]. Our lab observations made during video capture for this study revealed that cicadas display diverse flight modes like those exhibited by highly maneuverable insects, for example Odonates, despite cicadas being more massive [29], which indicates the capability of cicada-inspired MAVs to carry heavier payloads (in terms of body mass). This motivates quantification of cicada wing and body kinematics, as well as the aerodynamics that are appropriated by cicadas to perform more advanced maneuvers.

Zeyghami et al. reported that when performing maneuvers, despite having different body orientations and motions in forward flight, turns, and climbing flight, cicadas use a similar force control strategy. The cicadas they studied did not vary the stroke plane relative to their bodies considerably, and the local orientation of flight forces was similar among the flight modes [28]. Less stroke plane variation points to fewer degrees of freedom of the wing, and hints at a simpler wing actuation apparatus [28,30,31,32]. To induce maneuvers when the stroke plane is constrained relative to the body, flying animals use changes in body posture to reorient the stroke plane in the global frame [7,25,33]. Considering the limited range of stroke plane motion relative to the body in other flight modes, we predict that the cicadas in the present study will primarily rely on body postural changes for backward flight. Nevertheless, we do not know whether or how the cicadas will vary the orientation of flight forces relative to the body during backward flight in comparison to other previously identified flight modes.

To understand the kinematics and aerodynamics trends of backward flight, we used a combination of high-speed photogrammetry and three-dimensional reconstruction to capture the flight kinematics. A computational fluids dynamics (CFD) solver was then employed to calculate flight forces and visualize the flow features. The cicadas we observed were either engaged in free flight shortly after takeoff before switching the flight mode to backward flight, or initiated backward flight directly from takeoff. All flights were self-motivated. The exact roles of the upstroke (ventrodorsal stroke) and downstroke (dorsoventral stroke) in force production in the local and global frame, the force control strategy, and the aerodynamics during the backward flight of cicadas have not been elucidated before now and may not be direct extrapolations from forward flight.

## 2. Materials and Methods

### 2.1. Insects, Data Acquisition, and Three-Dimensional (3D) Surface Reconstruction

**Figure 1:**
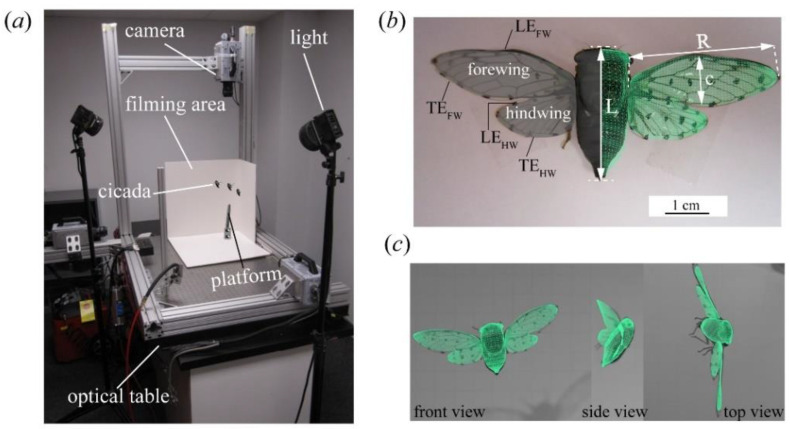
The cicada in free flight. (**a**) Experimental setup showing the filming arrangement with high-speed cameras. (**b**) Cicada (*Tibicen linnei*) image and template (shown in green) with relevant labels. LE—leading edge, TE—trailing edge, FW—forewing, HW—hindwing, C is the mid-span chord, L is the body length, R is the wing length. (**c**) Reconstructed cicada template overlapped on the cicada in free flight.

The methods used in this work have been documented in previous works [14,15,34] and are briefly outlined here. Cicadas (both *Tibicen linnei* (annual species) and *Magicicada septendecim* (seventeen-year periodical species)) were captured in the wild and transported to the lab for experiments. Afterward, we dotted their wings for tracking purposes using a felt-tip marker. The insects were then placed on a platform, where their voluntary flight was captured using three synchronized orthogonally arranged high-speed cameras (Photron FASTCAM SA3, Photron USA, Inc., San Diego, CA, USA) recording at 1000 frames per second (Figure 1a). Of the captured footage, which included similar flights to those observed in previous works [14,15,28], we obtained two backward flight sequences that were of a substantial in-flight duration. Some of these videos are supplied in the electronic Supplementary Materials (Video S1, S2, and S3). The cicadas either transitioned into backward flight after a few wingbeats (CCD #1, Video S1) or initiated backward flight directly from takeoff (CCD #2, Video S2). Using a template-based reconstruction technique [34] (Figure 1b,c), we obtained a 3D model of the cicada, in which the body is a solid surface and the wings are membranes. The modeling process was performed in Autodesk Maya. Each surface is composed of Catmull–Clark subdivision surfaces, which are spline representations of a mesh topology; therefore, these surfaces are suitable to model the deformable solid and membrane surfaces of the body and wings [35]. At each time frame, subdivision surface nodes on the digital wings were modified in a point-by-point manner to match those of marked points on the cicada wing (see Figure 1b, left side of the cicada) so that the digital wing was precisely aligned with the position and deformation of the real cicada’s wing (see Figure 1c). Body motion was generated in a similar manner, although the body motion was slower than that of the wings and was therefore easier to track. The Catmull–Clark surfaces were then modified using small unstructured triangular subdivisions (see Figure 1b, right side of the cicada) that refined the original surface mesh. The motion of the body and wings, as captured using this technique, was used for kinematics analysis and CFD simulations. The morphological parameters of the two selected cicadas are documented in Table 1.

### 2.2. Wing Kinematics Definitions

**Figure 2:**
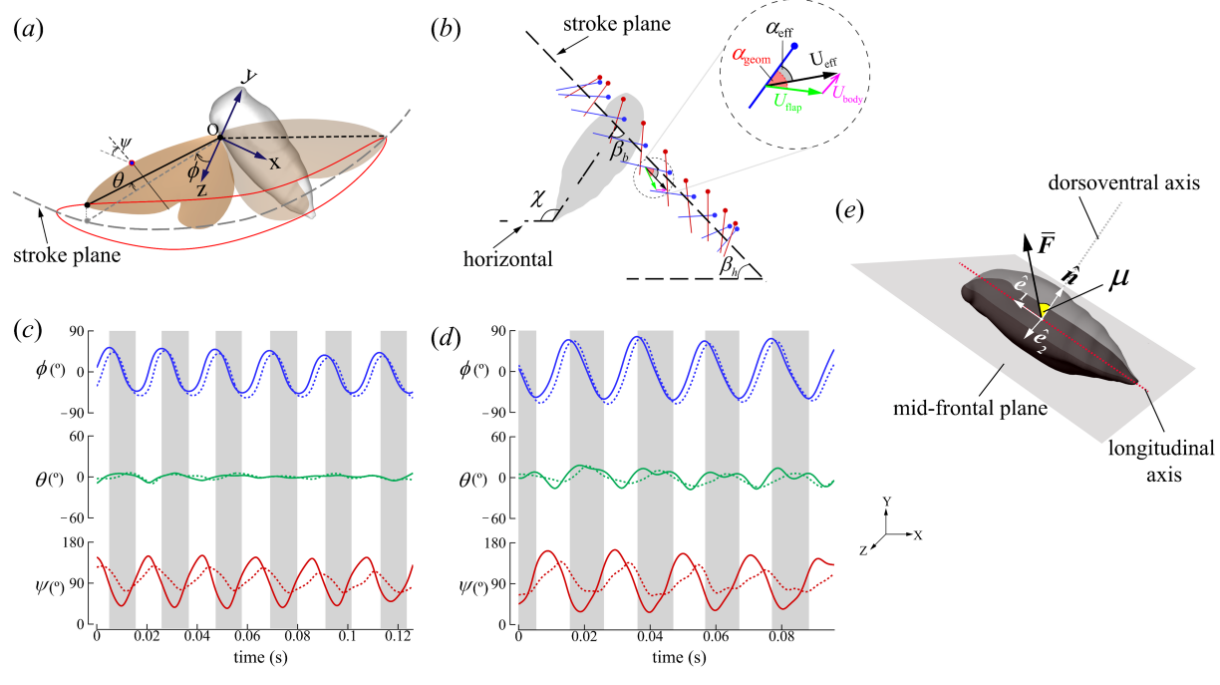
Relevant definitions. (**a**) Wing Euler angle definitions. (**b**) Wing chord at 0.75R. US—blue, DS—red. Measured wing kinematics of (**c**) CCD #1 and (**d**) CCD #2 based on the definitions in (**a**). The solid and dashed lines represent the forewing and hindwing measurements, respectively. (**e**) ${\hat{e}}_{1}$, ${\hat{e}}_{2}$, and $\hat{n}$ are are orthonormal and form the basis of the local/body coordinate frame. The angle between the half-stroke-averaged aerodynamic force $\left(\bar{F}\right)$ and body normal $\left(\hat{n}\right)$ is denoted as *μ*, X, Y, and Z form the basis for the global coordinate frame.

From the reconstruction, we quantified the wing kinematics. A coordinate system was fixed at the wing root, and the kinematics were measured with respect to the mean stroke plane. The stroke plane was defined as the least-squares reference plane that passed through the centroid of the points of the wing root and tip coordinates. We averaged the stroke plane for all complete wings beats to obtain the mean stroke plane. The Euler angles of flap (ϕ), deviation (θ), and pitch (ψ) define the rigid wing orientation relative to the stroke plane (Figure 2a). ϕ refers to the forward and backward motion of the wing projected on the stroke plane. The up and down rotation with respect to the mean stroke plane is expressed by θ. ψ is the angle between the wing chord and the mean stroke plane. ψ_DS_ is less than 90° and ψ_US_ is greater than 90° in our definition. The geometric angle of attack (AoA) (α_geom_) is the angle between the wing chord and flapping velocity, while the effective AoA (α_eff_) is the angle between the wing chord and the vector sum of the body and wing velocities (Figure 2b).

### 2.3. Numerical Methods and Simulation Setup

A CFD simulation is a direct numerical simulation (DNS) based on a sharp-interface immersed boundary flow solver for simulating incompressible flows around 3D objects [36]. Example studies demonstrating the capability of this solver to simulate complex biological boundaries include butterfly, dragonfly, and hummingbird flights [24,25,37]. Validations can be found in previous works too [14,15,38]. The methods are outlined here. The governing equations in this work use the time-dependent incompressible viscous Navier–Stokes (N–S) equation (Equation (1)):

(1) $$\nabla \cdot u\;=\;0;\;\frac{\partial u}{\partial t}+u\cdot \nabla u=-\frac{1}{\rho }\nabla p+\upsilon \nabla^{2}u$$

where u is the velocity vector, ρ is the density, υ is the kinematic viscosity, and p is the pressure. The N–S equation was discretized on non-conforming cartesian grids and boundary conditions on the immersed boundary were imposed using a ghost-cell procedure. Time stepping was achieved using the fractional step method [39] to decouple pressure and velocity terms while solving Equation (1). The momentum equation was first solved to obtain intermediate velocities. The second-order Adams–Bashforth scheme and implicit Crank–Nicolson scheme were used to discretize the convection and diffusion terms of the momentum equation, respectively. To enforce the incompressibility constraint, the intermediate velocity field was projected through a divergence-free vector field, necessitating the solution of the pressure Poisson equation (PPE). The PPE was discretized via the second-order center difference scheme and solved using a fast multigrid (MG) method with an implicit smoother. The MG method reaches a converged pressure field with rapid decay in the residuals [40]. Finally, pressure gradients were used to update the intermediate velocities, yielding a divergence-free velocity field.

**Figure 3:**
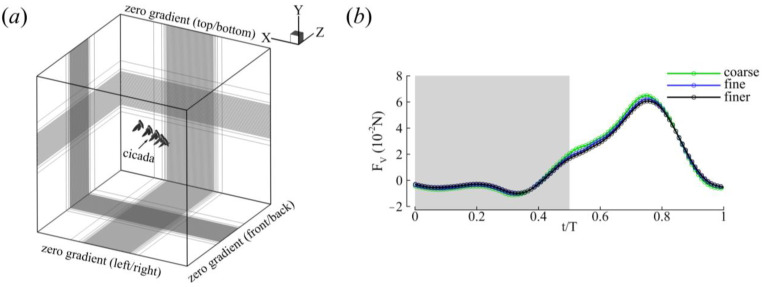
CFD simulation setup. (**a**). Computational domain with boundary conditions. For display, the meshes were coarsened 9, 6, and 3 times in the x, y, and z directions, respectively. (**b**) Grid refinement. The vertical force during the second flapping stroke of CCD #1 is shown. Gray shading denotes the DS. ‘Fine’ grids are shown in (**a**).

The domain boundary conditions of both the pressure and velocity were zero gradients. The size of the computational domain was 50 c × 50 c × 50 c (Figure 3). The Reynolds number, defined as $Re=\frac{{\bar{U}}_{eff}c}{\upsilon }$, is measured based on the FW mid-span chord length (*c* = 0.014 m), the kinematic viscosity of air at room temperature (υ = 1.5 × 10^−5^ m^2^/s), and the average effective wing tip speed (${\bar{U}}_{eff}=\frac{1}{T}\int_{0}^{T}\sqrt{({\dot{x}}_{tip}+{\dot{x}}_{body}{)}^{2}+({\dot{y}}_{tip}+{\dot{y}}_{body}{)}^{2}+({\dot{z}}_{tip}+{\dot{z}}_{body}{)}^{2}}dt$, where $\left\langle \dot{x},\dot{y},\dot{z}\right\rangle$ is the time derivative of the displacement vector and *T* is the flapping duration), and ranged between 5400 and 9300 for both cicadas. These *Re* values are in the expected range for large insects. The vortex structures were visualized using positive values of the Q-criterion [41] (Equation (2)):

(2) $$Q=\frac{1}{2}\left[{\left|\Omega \right|}^{2}-{\left|S\right|}^{2}\right]>0$$

where $S=\frac{1}{2}\left[\nabla u+{\left(\nabla u\right)}^{T}\right]$ and $\Omega =\frac{1}{2}\left[\nabla u-{\left(\nabla u\right)}^{T}\right]$ are the strain rate and vorticity tensors, respectively. A grid convergence study was set up based on different mesh sizes (Figure 3b and Table 2). The simulation results presented are based on the ‘fine’ grid results. The difference between the means as well as the maximum values of the fine and finer grids was about 2% (Table 2); thus, the fine mesh was deemed sufficient for the current study.

## 3. Results

### 3.1. Kinematics

#### 3.1.1. Body Kinematics

**Figure 4:**
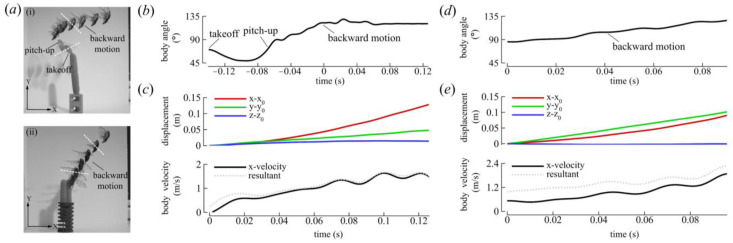
Body kinematics. (**a**) Montage of flight sequences of (**i**) CCD #1 and (**ii**) CCD #2. The transparent cicadas in (**i**) denote the flight phases preceding backward flight (takeoff and pitch-up) of CCD #1. The white dashed lines in (**i**,**ii**) qualitatively denote the stroke plane orientation. (**b**) Body angle and (**c**) center of mass displacements and velocity of CCD #1. (**d**) Body angle and (**e**) center of mass displacements and velocity of CCD #2.

The body kinematics of the selected cicadas are shown in Figure 4. CCD #1 initiated flight voluntarily and flew upward and forward during the preparatory phase, as indicated by the transparent images in Figure 4a(i). Afterward, the cicada pitched its body to a steep angle, slowed down, and initiated backward flight. The cicada flew for approximately six flapping strokes in a relatively straight path with a mean body angle ($\bar{\chi }$) of 122° before leaving the view of one the cameras (Figure 4a(i),b). The average backward velocity (${\bar{U}}_{b}$) was −1 m/s as the insect flew in a flight angle of about 15° relative to the horizontal plane (Figure 4c). CCD #2 also initiated flight voluntarily, albeit via a jumping takeoff. Its initial body angle was 86°, which increased to 130° by the end of the flight (Figure 4d). This cicada flew for approximately four flapping strokes with a ${\bar{U}}_{b}$ of −0.94 m/s and increased its altitude by an angle of about 50° relative to the horizontal plane (Figure 4e). The advance ratio (*J*), which is defined here as the ratio of the average resultant body velocity to the wingtip velocity, was about −0.2 for both cicadas.

#### 3.1.2. Wing Kinematics

**Figure 5:**
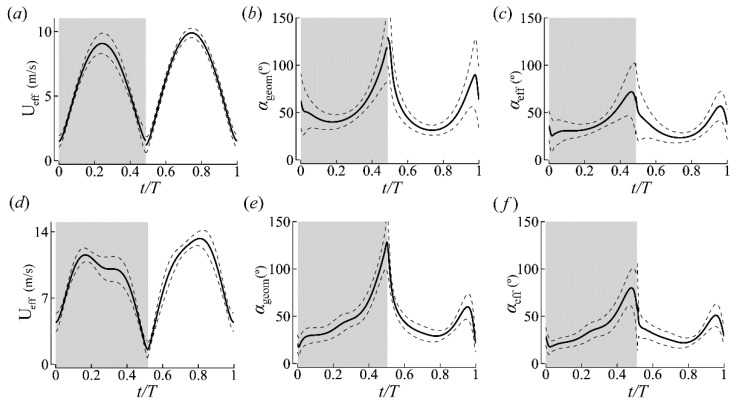
Additional forewing kinematics parameters. (**a**) Effective wing tip speed, (**b**) geometric AoA at 0.75R, and (**c**) effective AoA at 0.75R for CCD #1. (**d**–**f**) CCD #2′s data. Solid and dashed lines represent the mean ± standard deviation of all of the complete wingbeats, respectively. Gray shading denotes the DS.

The average stroke plane kinematics of the left and right wings are reported in Figure 5 and summarized in Table 3. Although the FW and HW of the cicada were functionally coupled, the FW led the HW with a slight phase difference (<25°), corroborating field observations of cicadas in forward flight [42]. For CCD #1, the wing pairs (FW and HW) traversed a stroke plane inclined at 73 ± 2° relative to the longitudinal axis of the body ($\beta_{b}$). The stroke plane angle relative to the horizontal ($\beta_{h}$) was 46 ± 3° (see Figure 2b for definitions). The stroke amplitude (Φ) was 92 ± 5° and similar for both wing pairs, although the average pitch angles (ψ) of the HW were larger. The HW’s rotation also lagged behind the FW’s, which is similar to Lepidopterans with functionally coupled wings [43]. The DS-to-US duration ratio (DS/US) was 0.95, and the DS-to-US ratio of the average effective tip velocity squared ${\bar{(U}}_{DS}/{\bar{U}}_{US}{)}_{eff}^{2}$ was 0.88. The time histories of the angles of attack (AoAs) at 0.75R of the FWs are shown in Figure 5. $\alpha_{geom}$ was 56 ± 5° and 53 ± 7° in the DS and US, respectively. $\alpha_{eff}$ was 41 ± 7° and 36 ± 5° in the DS and US, respectively. Discontinuities in the wing kinematics are attributed to computing the half-stroke averages.

For CCD #2, $\beta_{b}$ and $\beta_{h}$ were 69 ± 2° and 37 ± 13°, respectively. Similar to CCD #1, $\beta_{b}$’s variation was small, while $\beta_{h}$’s variation was more substantial due to changes in body angles over a greater range (86–130°) during flight (Figure 4d). Φ was 133 ± 5° for both the wing pairs and ψ was higher for the HWs. DS/US was 1.06, and ${\bar{(U}}_{DS}/{\bar{U}}_{US}{)}_{eff}^{2}$ was 0.83. $\alpha_{geom}$ was 52 ± 4° and 43 ± 4° in the DS and US, respectively. $\alpha_{eff}$ was 39 ± 5° and 32 ± 4° in the DS and US, respectively. In general, the DS’s AoA was higher than the US’s, while the wing US’s velocity was higher than the DS’s for both insects (Figure 5 and Table 3).

### 3.2. Aerodynamic Forces

**Figure 6:**
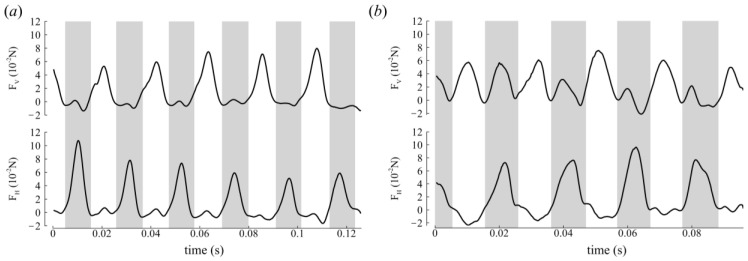
Time history of force production in the global frame of (**a**) CCD #1 and (**b**) CCD #2. F_V_—vertical force and F_H_—horizontal force refer to the forces in the Y and X directions, respectively (see Figure 2a). Gray shading denotes the DS.

From the CFD simulation, we obtained the aerodynamic forces by integrating the shear stress and pressure on the wing. Given the biologically accurate *Re* of the simulations, there exists high-frequency components of the force data that are smoothed out to clarify trends in the time history, which is a similar procedure to that performed in insect-like flapping-wing experiments [44,45]. Horizontal forces were produced in the DS for backward propulsion while vertical forces were produced in the US for weight support for CCD #1 (Figure 6a). In the first half of CCD #2′s flight (Figure 6b, t = 0–46 ms), both half strokes generated vertical and horizontal forces. The US produced vertical forces for weight support and horizontal forces that opposed the backward motion, while the DS generated vertical forces for weight support and horizontal forces that propelled the insect backward. This trend was probably due to the less steep $\beta_{h}$ (approximately 20°). Nevertheless, in the second half of flight (t = 46–96 ms), CCD #2′s force production trend was similar to CCD #1′s, whereby horizontal forces were produced predominantly in the DS while vertical forces were produced in the US for weight support. Here, $\beta_{h}$ increased to approximately 45°, which is similar to CCD #1′s $\beta_{h}$. The time-averaged vertical forces (${\bar{F}}_{V}$) were 1.3× bodyweight (BW) and 1.5× BW for CCD #1 and CCD #2, respectively, while the time-averaged horizontal forces (${\bar{F}}_{H}$) were 1.2× BW and 1.3× BW for CCD #1 and CCD #2, respectively, for all complete strokes.

#### Force Orientation in the Global and Local Frames

In Section 3.2, we quantified the magnitudes of the generated flight forces; however, the orientation of the flight forces is essential for positioning the insect in its intended travel direction. Here, we quantify the force orientation both in the global and local/body frames to formulate an enhanced understanding of cicada backward flight mechanisms. A simple technique for orienting flight forces involves tilting the stroke plane [17,46], which is achieved either (i) through actuation from the wing hinge to rotate the stroke plane relative to the body (here, the body angle changes slightly), or (ii) through reorienting the body angle or posture while the stroke plane relative to the body is fixed with slight changes within a narrow range. The latter (ii), known as force vectoring, is employed to reorient flight forces for maneuvers when the aerodynamic force is constrained within the animal’s body frame [2,28,33,46].

CCD #1 $\beta_{b}$ was relatively fixed during flight (Table 3) and Figure 4a illustrates that the stroke plane in the global frame was reoriented by changes in the body angle. The difference in body angle before and after backward flight initiation was ~80° for CCD #1 (Figure 4b). CCD #2 also maintained a steep body posture from takeoff. When the body angle was low, the stroke plane was oriented more downward. Conversely, when the body angle increased, the stroke plane was oriented upward (Figure 4a(ii)). The reorientation of the stroke plane due to the body angle is likely the major influence on the orientation of the force vector in the global frame.

**Figure 7:**
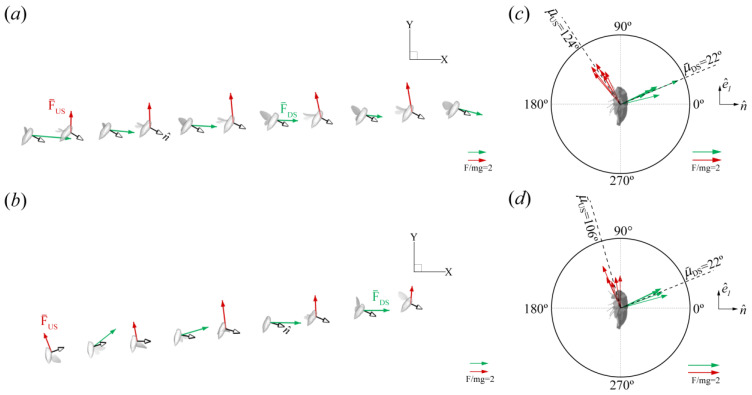
Force orientation in the global and local frames. (**a**,**b**) Half-stroke-averaged forces of CCD #1 and CCD #2, respectively, in the global frame. Red and green arrows represent ${\bar{F}}_{US}$ and ${\bar{F}}_{DS}$, respectively. The force vectors have been superimposed on the cicada at midstroke. For illustration purposes, the real spacing between each cicada model in the X-direction has been scaled up by ten chord lengths. The vector orientation, as well as the spacing in the Y-direction, were unaffected. (**c**,**d**) Orientation of the force vector relative to the body projected on the mid-sagittal plane of CCD #1 and CCD #2, respectively. μ = 0° when $\bar{F}$ is aligned in the same direction as $\hat{n}$.

In the global frame, the half-stroke-averaged aerodynamic force vectors presented are in the X-Y plane, where most of the body motion occurred (Figure 7a,b). The green arrows and red arrows represent the DS average (${\bar{F}}_{DS}$) and US average force vectors (${\bar{F}}_{US}$), respectively. The US forces point upward (+Y direction), while the DS forces point backward (+X direction). Measured relative to the horizon (+X direction, Figure 2), ${\bar{F}}_{DS}$ and ${\bar{F}}_{US}$ were oriented at −6 ± 4° and 96 ± 5° for CCD #1, and at 14 ± 18° and 97 ± 10° for CCD #2, which indicates, in the global frame, predominantly vertical force generation during the US and horizontal force generation during the DS.

The orientation of the forces relative to the cicada body (local frame) was obtained by calculating the angle (μ) between $\bar{F}$ and $\hat{n}$ (Figure 2b,d). $\hat{n}$, which always points outward from the body, is perpendicular to the longitudinal axis vector (${\hat{e}}_{1}$) and represents the dorsoventral axis of the body (Figure 2d). ${\bar{\mu }}_{DS}$ and ${\bar{\mu }}_{US}$ were 22 ± 5° and 124 ± 5° for CCD #1, and 22 ± 5° and 106 ± 10° for CCD #2. $\bar{F}$ was produced only on the anterior side of the body, which is defined as the half-disk from 0° to 180°, counterclockwise (Figure 7b,d). The forces in the dorsoventral stroke (downstroke in the body frame [7]) were produced on the dorsal side (half-disk from 90° to 270°, clockwise), with the major component pointing in the dorsoventral axis direction. The forces produced in the ventrodorsal stroke (upstroke in the body frame [7]) were produced in the ventral side (half-disk from 90° to 270°, counterclockwise), with the major component pointing in the longitudinal axis direction. The variation in the orientation of the mean force vector relative to the body (±10°) was within the range reported for other organisms, as well as helicopters (±20°), which use force vectoring [33].

### 3.3. Three-Dimensional Wake Topology and Leading-Edge Vortex Strength

**Figure 8:**
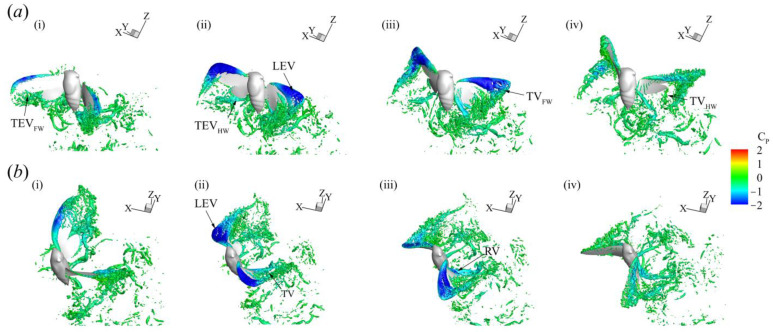
Flow structures visualized using the Q-criterion (Q = 600) and colored according to the pressure of the vorticial structures during the third flapping stroke of cicada #1 (t = 57–80 ms). (**a**) Top row (**i**–**iv**) represents snapshots during the DS at t/T = 0.13, 0.25, 0.38, and 0.48, respectively. (**b**) Bottom row (**i**–**iv**) denotes snapshots during the US at t/T = 0.63, 0.75, 0.88, and 0.98. The flow is colored according to the coefficient of pressure $C_{p}=(p-p_{\infty })/0.5\rho {\bar{U}}_{eff}$. TEV—trailing-edge vortex; TV—tip vortex; RV—root vortex.

Here, we visualized the flow around the insect using the iso-surface of the Q-criterion (§2.3.) to understand how flight forces were produced. The evolution of the flow features throughout a representative stroke of CCD #1 (t = 57–80 ms) is displayed in Figure 8 and colored according to the coefficient of pressure. The video can be found in the ESM (Video S4).

Large coherent structures with strong vorticity around the wing’s surface were identified. An LEV (a region of low pressure, shown in blue) was formed shortly after the half strokes (Figure 8a(i),b(i)) and remained attached for the duration of each half stroke, shedding at the stroke reversal. The size of the LEV in both half strokes was similar, qualitatively indicating that comparable amounts of force were generated which is corroborated by the force measurements shown in Figure 6. Other vortex structures such as a trailing-edge vortex, tip vortex, and root vortex were also evident. Most of the large vortex structures emanated from the FWs, which may indicate the auxiliary role that the HWs play in force production during flight.

**Figure 9:**
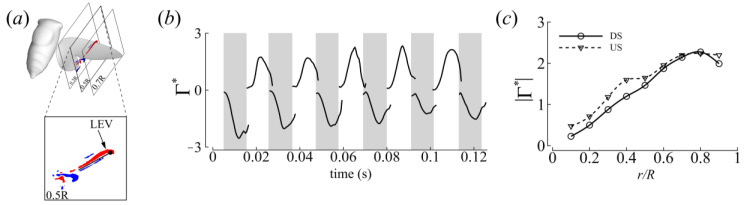
LEV circulation calculated for CCD #1. (**a**) Calculation of LEV circulation. (**b**) Time history of LEV circulation at the mid-span (0.50R). (**c**) Mean spanwise distribution of circulation at mid-stroke for all complete strokes.

To measure the strength of the LEV, two-dimensional (2D) planes perpendicular to the rotation axis of the LEV were placed along the wing at every time step of the numerical simulation (Figure 9a). The vorticity (ω) was calculated on this 2D plane by taking the curl of the velocity. The area of integration (*S*) was identified by a vorticity threshold set at 10% of the maximum spanwise vorticity. Subsequently, the non-dimensional circulation was obtained using Equation (3).

(3) $$\Gamma_{LEV}^{*}=\frac{1}{c{\bar{U}}_{eff}}\iint_{S}\omega \cdot dS$$

Both the time histories and spanwise distribution of the circulation are reported in Figure 9b,c. Consistent with the force measurements, substantial LEV circulation was recorded in both half strokes. Averaged across all strokes, the DS-US LEV circulation ratio was about 1 (Table 4), quantitatively indicating similarity in the vortex forces generated during half strokes.

### 3.4. Roles of the Forewing and Hindwing

**Figure 10:**
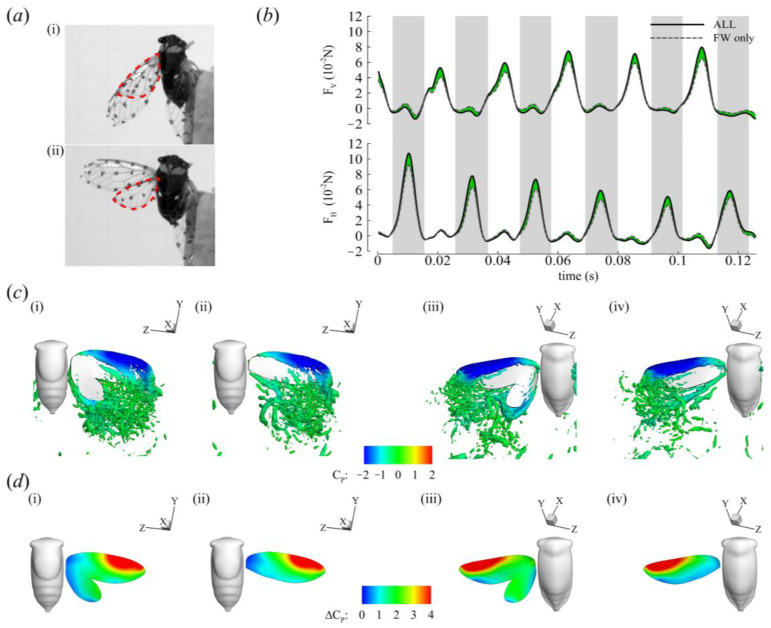
Forewing and hindwing force generation (**a**). (**i**) Wing configuration before flight. The HW (outlined with red dashed lines) is tucked under the FW. (**ii**) Wing configuration in flight. The HW leading edge is connected to the FW trailing edge. (**b**) Force production of CCD #1. Gray shading denotes the DS. (**c**) Flow structures at the mid-DS (**i**) when the HW is present versus (**ii**) when the HW is absent, and at the mid-US when the HW is (**iii**) present versus (**iv**) absent. (**d**) Pressure differences on the wing surface at exact snapshots are shown in (**c**).

Cicadas have two pairs of wings, of which the HW pair is the smaller. The HW is attached to the FW in flight to form a functional two-winged flight apparatus (Figure 10a). Functionally coupling the FW and HW together is thought to eliminate the conflict between the wing pairs [47]. Here, we quantified the contribution of each wing pair to force generation. We compared two simulation cases: (i) ALL (FW+HW) and (ii) FW only (FO). An HW only (HO) case was not simulated because the flow cannot separate at the leading edge of the HW where it is connected to the FW’s trailing edge.

The time history of the forces of CCD #1 is presented in Figure 10b (see Figure S1 in Supplementary Materials for the time history of CCD #2). The stroke-averaged net force of the ALL case was about 22% greater than the FO case for both cicadas, with most of the difference between the two cases occurring in the mid-stroke region. The presence of the HW did not significantly affect vortex formation on the FW (Figure 10c). This observation is corroborated by plots of the pressure difference between the top and bottom surfaces of the wings during the mid-stroke (Figure 10d(i–iv)) for both the ALL and FO cases. Regions of high pressure differences (shown in red) correspond to where the LEV resides and indicate where most of the force is produced during both half strokes. This region was similar in size for both the ALL and FO cases. The presence of the HW, however, may have influenced the pressure distribution around the trailing edge (TE) of the FW, as indicated by the expansion of the high pressure difference region to the TE of the ALL case compared to the FO case (Figure 10d(i–iv)). Most of the force contribution from the HW comes from the distal part of the HW, where it is no longer connected to the FW and the velocity is highest [15] (Figure 10d(i)). The HW forces are not enough for weight support but the HW may be more important for other functions such as evasiveness or turning in flight as seen in functionally two wings flies with well-developed HWs [48].

## 4. Discussion and Conclusions

In this study, we investigated a new flight mode of the cicada, i.e., backward/reverse flight, which is an avenue of additional maneuverability for this heavy flier. We studied the coordination between the wing and body motion in connection with the production and orientation of aerodynamic forces. Here, our findings are further discussed and compared to previous research.

Cicadas typically fly forward with body angles of ~10–50° with the stroke plane inclined downward [14,49]. During backward flight, however, the stroke plane was tilted upward and χ was large, ranging between 86 and 130° (Figure 4 and Table 5). Also, the χ value recorded for the cicada in backward flight was larger than those previously measured for the backward flights of hummingbirds (50–75°) [10], dragonflies (85–95° [25]; 100° [3]), waterlily beetles (50–70°) [5], and cockchafer beetles (87–115°) [26]. A steep body posture and an upward-tilted stroke plane are common features known thus far of backward flight between Pterygota and hummingbirds and are probably techniques shared due to convergent evolution, as previously suggested [10]. This tilted stroke plane is also observed in butterflies [24]. However, unlike hummingbirds, which tilt their stroke plane upward and flatten it relative to the horizontal during backward flight [10], both cicadas and dragonflies maintain a steeply inclined stroke plane in backward flight just as they do during forward flight (Table 6) [25].

A possible consequence of the upright body posture appropriated during backward flight is an increase in drag. However, if the body angle is very steep, the drag contribution of the body may not be considerably different from forward flight at similar angles relative to the incoming flow. For instance, by maintaining a steep body angle of ~122° (CCD #1), the flow that the body experiences is equivalent to orienting the body at 58° in forward flight and is not considerably different from flying at 50°, which is the upper range of body angles used during cicada forward flight. Using a hummingbird body model without wings in a wind tunnel, Sapir and Dudley showed that although drag during backward flight was higher, it only differed by 3.6% from forward flight although the body angle difference was 33°, that is, an 88% increase in body angle [10]. Bode-Oke et al. also found that the parasitic drag (viscous + pressure) coefficient in dragonfly backward flight was in the range measured for the forward flight of dragonflies in wind tunnels at similar *Re* values [25]. The parasitic drag coefficient is defined as ${\bar{C}}_{D}={\bar{F}}_{H}/0.5\rho {{\bar{U}}_{b}}^{2}S_{frontal}$, where ${\bar{F}}_{H}$ is the mean body horizontal force (and is less than 5% of the horizontal forces produced by the wings), $S_{frontal}=A_{MF}sin(\bar{\chi })$ is the frontal area, and $A_{MF}$ is the cross-sectional area in the mid-frontal plane. ${\bar{C}}_{D}$ was 1.18 for CCD #1, which was higher than that reported in [15] (0.52). High body drag may be inevitable if force vectoring is the only mechanism by which cicadas elicit backward flight. Typical backward flight speeds are about 1 m/s for dragonflies [24] and DelFly II MAV [50], 1.5 m/s for bumblebees [51], and 2 m/s for hummingbirds [10]. At these low flight speeds, drag penalties are not as critical [7,17].

**Table 5:** Forward versus backward flight of cicadas. $\bar{F}$ is the resultant force normalized by the body weight during each half stroke, while ${\bar{F}}_{V}$ is the component of the resultant force that solely contributes to weight support.

Flight Mode	*J*	${\bar{U}}_{b}$ (m/s)	$\bar{\chi }$ (°)	$\beta_{b}$ (°)	$\beta_{h}$ (°)	DS/US	$\alpha_{eff}$ (°)		${\bar{F}}_{DS}$	${\bar{F}}_{US}$	${\bar{F}}_{V,DS}$	${\bar{F}}_{V,US}$	Reference
							DS	US					
Forward	--	1.9	25	--	--	--	--	--	--	--	--	--	[52]
	0.32	2.0	28	64	36 *	1.0	--	--	3.33	0.53	3.23 ^‡^	0.10	[15]
	0.32	2.2	49	62	13 *	1.17	62	72	1.91	0.59	1.58 ^‡^	0.47	[14]
Backward	−0.19	−1.0	122	73	46 ^†^	0.95	41	36	2.89	2.96	−0.28	2.87 ^‡^	current study
	−0.17	−0.94	107	69	37 ^†^	1.06	40	34	3.14	2.35	0.81	2.27 ^‡^	

*—stroke plane tilted downward relative to the horizontal plane, ^†^—stroke plane tilted upward relative to the horizontal plane, ^‡^—predominant weight-supporting half stroke.

The slight variation in the mean stroke plane relative to the body (<±5°) observed in the kinematics results indicates that cicadas do not considerably control this angle, possibly due to a limited range of joint rotation. $\beta_{b}$ was inclined at approximately 70° for both cicadas and was marginally larger than the values measured in forward flight by about 10°, signifying that a small stroke plane tilt away from the body occurred in backward flight (Table 5). However, this slight tilt alone did not induce backward flight. Instead, the reorientation of the body in the global frame caused the major reorientation of the force vector in the global frame compared to forward flight (refer to the illustration in Figure 7a). The horizontal motion of the cicadas occurred from left to right (in the +X direction; Figure 4a). Measured relative to the horizon using the components in the mid-sagittal plane, ${\bar{F}}_{DS}$ and ${\bar{F}}_{US}$ were oriented at −6 ± 4° and 96 ± 5° for CCD #1 and 13 ± 18° and 97 ± 10° for CCD #2, respectively. Likewise, ${\bar{F}}_{DS}$ and ${\bar{F}}_{US}$ were oriented at 91° and 16°, respectively, during forward flight [15]. Our findings indicate that the orientations of force vectors in the DS and US in backward flight were reversed relative to forward flight in the global frame. Comparing the final body orientations of the cicadas in forward and backward flight moving in the same direction (+X direction), it is as though the DS force vector in forward flight was rotated clockwise by 78–97° while the US force vector was rotated counterclockwise by about 80° simply by changing the body angle. This reorientation of the force vector also reversed the aerodynamic functions of the DS and US in the global frame. In forward flight, the DS and US predominantly provide weight support and forward thrust, respectively, whereas in backward flight, the DS and US mainly provide backward thrust and weight support, respectively (Figure 7).

It is also relevant to compare the wing pitch kinematics (ψ) of backward flight to those of forward flight. Given the dominant contribution of the FW to the generation of major wake structures (Figure 10), we focus on the FW kinematics. In forward flight, the pitch of the FW was measured to range between 44 and 133° [14] and from 53 to 112° [15] (in the downstroke and upstroke, respectively). In the current backward flight investigation, we found that the wing pitch has a larger amplitude (CCD #1 ranged from 44 to 150° in the ‘backward flight’ sequence; CCD #2 ranged from 27 to 168° in the ‘backward flight’ sequence), further emphasizing the role reversal of the DS and US in backward flight. CCD #1′s US pitch angles during backward flight were considerably higher than those found in forward flight. Considering the body is inclined past the vertical axis, the larger FW pitch results in HW surfaces that are closer to being horizontal in the global plane and therefore are more suitably oriented for vertical force generation in the US. CCD #2 did not start its backward flight with as steep of a body angle as CCD #1 (Figure 4b,d); however, the increased US pitch angle resulted in a similar FW surface orientation.

Because the US carries the insect’s weight in backward flight, the aerodynamic demand on the US increases, and it has to become more aerodynamically active when compared to forward flight (see ${\bar{F}}_{V,US}$ in Table 5, for example). Therefore, in the global frame, the half-stroke aerodynamic function was not only reversed, but the force magnitude was also influenced. Although the cicadas in this study were accelerating, if they had approached cruising, where the wing horizontal forces are balanced by body drag, it is expected that the horizontal force magnitude, which is generated in the DS in backward flight, would have decreased [53,54]. Consequently, the US-DS force asymmetry would become more pronounced. Also, assuming similar the net output from the wings during cruising flight in the forward or backward direction, as well as a fixed stroke plane relative to the body, it becomes clearer that the body angle modulates the distribution between vertical and horizontal forces in the global frame.

**Table 6:** Kinematics of backward flight among different fliers. DS/US, $\alpha_{eff}$, and $\bar{F}$ are split by the contribution of the forewing and hindwing, respectively, for functionally four-winged insects. $\alpha_{eff}$ is reported at 0.75R.

Animal	− *J*	${\bar{U}}_{b}$ (m/s)	χ (°)	$\beta_{b}$ (°)	$\beta_{h}$ (°)	DS/US		$\alpha_{eff,DS}$ (°)		$\alpha_{eff,US}$ (°)		${\bar{F}}_{DS}$		${\bar{F}}_{US}$		Reference
**Cockchafer beetle**	--	−1.2	87–115	--	--	--		--		--		--		--		[26]
**Hummingbird**	0.3	−1.5	51–75	57–71	−15–6	0.88–1.08		--		--		--		--		[10]
**Dragonfly**	0.3	−1.0	85–95	35	47	0.87	0.83	25	27	21	27	1.44	2.07	2.15	3.17	[25]
**Cicada**	0.2	−1.0	122	73	46	0.95		41		36		2.89		2.96		current study
	0.2	−0.9	86–130	69	37	1.06		40		34		3.14		2.35		
**Waterlily beetle**	--	--	50–70	40–50	0 –30	--		--		--		--		--		[5]
**Butterfly**	0.3–0.4	−0.4 to −0.8	85–119	78–86	0–35	0.82–1.45		53–72		33–51		1.3–2.1		1.3–2.6		[24]
**DelFly II**	--	−1.0	70–100	--	--	--		--		--		--		--		[54]

For insects that employ asymmetric strokes in an inclined stroke plane during hovering or forward flight, the US force, particularly for weight support, is minimal [15,16,17,18] (Table 7). The presence of an active US in backward flight suggests the presence of an LEV on the wing surface, which is essential at low *J* values and during accelerating flight (Figure 4). This is in addition to any enhancement of the US velocity due to the backward motion of the cicada. Here, the LEV was stably attached to the FW in both half strokes (Figure 8). Since the flow separates at the FW’s leading edge irrespective of the presence of the HW (Figure 10c), the FW forces were substantial due to vortex lift, which is a consequence of the LEV. Furthermore, when the HW was removed in the computational model, the LEV characteristics were not greatly influenced, corroborating previous results on revolving wings reporting that the FW morphologies match the formation of leading-edge vortices [38,55]. The measured DS-to-US LEV circulation ratio was about 1, indicating the presence of a strong LEV in the US for vertical force production. In past studies on cicada flight [14,15], the US LEV strength was much smaller than that of the DS.

**Figure 11:**
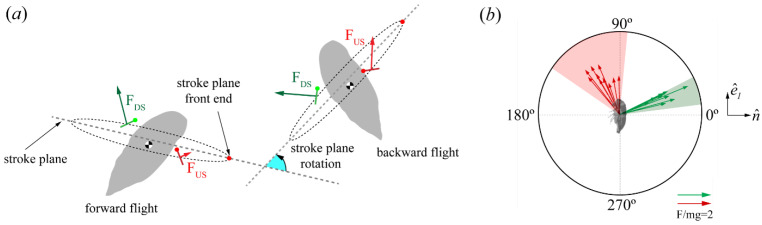
Force production and orientation in cicada flight. (**a**) Schematic illustrating the transition from forward to backward flight. (**b**) Orientation of the aerodynamic forces relative to the body normal. Data from previous research [14,15,28] are pooled together (shaded sectors on the circles). The arrows represent the data from the current study and are also shown in Figure 7.

Relative to the rest of the body, we showed that the flight forces were constrained in the anterior part of the body (Figure 7b,d). During the dorsoventral stroke (downstroke), the forces were directed with the major component pointing in the direction of the dorsoventral axis, whereas the ventrodorsal stroke (upstroke) was directed majorly in the longitudinal axis. In a 3D space, these force vectors form a cone whose axis is offset by $\bar{\mu }$ from the body normal (ventrodorsal axis), and the radii are expressed according to the standard deviation of all complete wing beats. ${\bar{\mu }}_{DS}$ and ${\bar{\mu }}_{US}$ were 22 ± 5° and 124 ± 5°, respectively, for CCD #1, while ${\bar{\mu }}_{DS}$ and ${\bar{\mu }}_{US}$ were 22 ± 5° and 106 ± 10°, respectively, for CCD #2. Prompted by previous work [28] and the drastic difference between the forward and backward flight body angles and stroke plane orientation in the global frame, we then explored whether cicadas used a unified force generation strategy (relative to the body) irrespective of the flight mode. ${\bar{\mu }}_{DS}$ and ${\bar{\mu }}_{US}$ ranged between 16 and 27° and between 85 and 135°, respectively, in previous work [14,15,28]. Figure 11b shows that the backward flight results fell within a similar range as the values recorded for forward flight and other flight modes.

Although the half-stroke-averaged aerodynamic forces are fixed in the same direction in the body frame, our findings do not suggest that the wing kinematics in all of these flight modes are the same. The wing kinematics vary according to the demand for force production or directional changes via torque generation. For instance, roll, pitch, or yaw torques can be generated by varying the wing AoA and the wing position relative to the body [28,37]. Additionally, the contribution of the US to the total force production increases in backward flight compared to a forward flight scenario due to US weight support. Increasing the US magnitude to accommodate weight support in the global frame indicates an increase in the ventrodorsal stroke’s force magnitude (in the body frame). Since the direction of the forces relative to the body is consistent in each half stroke for all flight maneuvers, the dorsoventral (downstroke) and ventrodorsal (upstroke) stroke function are not reversed in the body frame. Nevertheless, the force magnitude is modulated due to the demands of force production that ensure sustained flight in the global frame.

In the context of the work presented here, our understanding of cicadas can now be extended to backward flight. We have shown that cicadas perform backward flight by changing their body posture to reorient both the stroke plane and the force vector in the global frame. We found that their steep body posture also influenced the wing aerodynamics by reversing the aerodynamic roles of the half strokes compared to forward flight in the global frame. However, the orientation of aerodynamic forces relative to the body compared to other flight modes remained relatively fixed, despite significantly different body orientations and motions. An aerodynamically active upstroke signified by the presence of an LEV and high wing velocity was identified and the upstroke was principally responsible for weight support during backward flight. The LEV was present on the FWs, which generated most of the flight forces in comparison to the smaller HWs. Our results also clarify what the aerodynamics and kinematic adjustments may look like for other simple fliers (with a limited range of stroke plane motion relative to the body), such as beetles [5,26], which appropriate backward flight for both obstacle avoidance and interfacial flight, as well as MAVs, which may use backward flight during free flight or takeoff from vertical surfaces [50]. In addition to uncovering the strategies by which maneuverability could be obtained, future works should focus on the aerodynamic power consumed and inertial power required to perform backward flight, as efficiency is a key consideration for designing MAVs. MAVs inspired by cicadas may be desirable for their higher mass and simple stroke plane kinematics, and the results of this study provide guidance regarding how MAVs with cicada-like characteristics can achieve maneuverability through backward flight.

## Figures and Tables

**Table 1:** Morphological parameters of the selected cicadas. The levels of uncertainty for the mass and length measurements are ±1 mg and ±0.5 mm, respectively.

Species	ID	Body Weight (mg)	L (mm)	FW/HW Length (mm)	FW/HW Chord (mm)	FW/HW Area (mm^2^)	Flapping Frequency (Hz)
*Tibicen linnei*	CCD #1	1174	30	38/22	14/11	352/171	47.6
	CCD #2	1514	30	39/22	14/11	359/175	52.2

**Table 2:** Forces for the three different grid setups. Values are from the second flapping stroke of CCD #1.

	Grid Size	${\bar{F}}_{V}$ (10^−2^ N)	$F_{V,max}$ (10^−2^ N)
Coarse	336 × 216 × 192	1.48	6.50
Fine	480 × 320 × 216	1.45	6.22
Finer	600 × 392 × 336	1.42	6.08

**Table 3:** Summary of kinematic parameters for the backward flight of cicadas. The mean and standard deviation for all complete wing beats are documented here.

ID	*J*	${\bar{U}}_{b}$ (m/s)	$\bar{\chi }$ (°)	$\beta_{b}$ (°)	$\beta_{h}$ (°)	DS/US	${\left(\frac{{\bar{U}}_{DS}}{{\bar{U}}_{US}}\right)}_{eff}^{2}$	Φ (°)	$\alpha_{geom}$ (°)		$\alpha_{eff}$ (°)	
									DS	US	DS	US
CCD #1	−0.19	−1.0	122 ± 2	73 ± 2	46 ± 3	0.95	0.88	92 ± 5	56 ± 5	53 ± 7	41 ± 7	36 ± 5
CCD #2	−0.17	−0.94	107 ± 14	69 ± 2	37 ± 13	1.06	0.83	133 ± 5	52 ± 4	43 ± 4	39 ± 5	32 ± 4

**Table 4:** Half-stroke LEV circulation at the mid-span for CCD #1. ${\bar{\Gamma }}^{*}$ and ${\Gamma^{*}}_{max}$ represent the average and maximum circulation per half stroke, respectively. DS1 corresponds to the first gray shaded region in Figure 9b.

Half Stroke	${\bar{\Gamma }}^{*}$	${\Gamma^{*}}_{max}$	$\left|{{\bar{\Gamma }}^{*}}_{DS}/{{\bar{\Gamma }}^{*}}_{US}\right|$	$\left|{{\bar{\Gamma }}^{*}}_{max,DS}/{{\bar{\Gamma }}^{*}}_{max,US}\right|$
DS 1	−1.61	−2.58	1.62	1.46
US 1	0.99	1.76		
DS 2	−1.22	−2.07	1.18	1.20
US 2	1.03	1.72		
DS 3	−1.22	−1.94	0.98	0.85
US 3	1.25	2.28		
DS 4	−1.05	−1.80	0.90	0.76
US 4	1.17	2.36		
DS 5	−0.83	−1.27	0.70	0.59
US 5	1.18	2.16		
DS 6	−1.03	−1.55		

**Table 7:** Half-stroke force type and contribution to the resultant aerodynamic forces for several insects that use an inclined stroke plane in free flight. *—tethered.

Insect	Flight Mode	DS Force (%)	DS Force Type	US Force (%)	US Force Type	Reference
**Cicada**	forward	90	vertical	10	horizontal	[15]
		80	vertical	20	horizontal	[14]
	backward	49	horizontal	51	vertical	current study
		57	horizontal	43	vertical	current study
**Damselfly**	forward	84	vertical	16	horizontal	[18]
		75	vertical	25	horizontal	[56]
**Dragonfly**	hovering *	77	vertical	23	horizontal	[57]
	backward	33	horizontal	67	vertical	[25]
	forward	80	vertical	20	horizontal	[58]
		67	vertical	33	horizontal	[59]
**Fruit fly**	forward	61	vertical	39	horizontal	[60]
**Hawkmoth**	forward	80	vertical	20	horizontal	[61]
	hovering	67	vertical	33	horizontal	[62]
**Locust**	forward	84	vertical	14	horizontal	[63]
**Butterfly**	forward	74	vertical	26	horizontal	[64]
	backward	40	horizontal	60	vertical	[24]

## Data Availability

Data are contained within the article or Supplementary Materials.

## Supplementary Materials

The following supporting information can be downloaded at: https://www.mdpi.com/article/10.3390/biomimetics9040233/s1, Figure S1: CCD#2-Forewing versus hindwing force; Video S1: Cicada#1_backwardflight; Video S2: VideoS2_cicada#2_backwardflight; Video S3: VideoS3_Periodical cicada (*Magicicada* spp.) backward flight; Video S4: VideoS4_cicada#1-flow.

## References

[1] Sane S.P. (2003). The aerodynamics of insect flight. J. Exp. Biol..

[2] Dickinson M.H., Muijres F.T. (2016). The aerodynamics and control of free flight manoeuvres in Drosophila. Philos. Trans. R. Soc. B.

[3] Rüppell G. (1989). Kinematic analysis of symmetrical flight manoeuvres of Odonata. J. Exp. Biol..

[4] Rüppell G., Hilfert D. (1993). The flight of the relict dragonfly *Epiophlebia superstes* (Selys) in comparison with that of the modern Odonata (*Anisozygoptera*: *Epiophlebiidae*). Odonatologica.

[5] Mukundarajan H., Bardon T.C., Kim D.H., Prakash M. (2016). Surface tension dominates insect flight on fluid interfaces. J. Exp. Biol..

[6] Yokoi T., Fujisaki K. (2009). Hesitation behaviour of hoverflies *Sphaerophoria* spp. to avoid ambush by crab spiders. Naturwissenschaften.

[7] Dudley R. (2002). The Biomechanics of Insect Flight: Form, Function, Evolution.

[8] Lannoo M.J., Lannoo S.J. (1993). Why do electric fishes swim backwards? An hypothesis based on gymnotiform foraging behavior interpreted through sensory constraints. Environ. Biol. Fishes.

[9] Flammang B.E., Lauder G.V. (2016). Functional morphology and hydrodynamics of backward swimming in bluegill sunfish, Lepomis macrochirus. Zoology.

[10] Sapir N., Dudley R. (2012). Backward flight in hummingbirds employs unique kinematic adjustments and entails low metabolic cost. J. Exp. Biol..

[11] Collett M., Graham P., Collett T.S. (2017). Insect Navigation: What Backward Walking Reveals about the Control of Movement. Curr. Biol..

[12] Duysens J., Tax A.A., Murrer L., Dietz V. (1996). Backward and forward walking use different patterns of phase-dependent modulation of cutaneous reflexes in humans. J. Neurophysiol..

[13] Zeil J., Wittmann D. (1989). Visually controlled station-keeping by hovering guard bees of Trigona (Tetragonisca) angustula (Apidae, Meliponinae). J. Comp. Physiol. A.

[14] Wan H., Dong H., Gai K. (2015). Computational investigation of cicada aerodynamics in forward flight. J. R. Soc. Interface.

[15] Liu G., Dong H., Li C. (2016). Vortex dynamics and new lift enhancement mechanism of wing–body interaction in insect forward flight. J. Fluid Mech..

[16] Wang Z.J., Russell D. (2007). Effect of forewing and hindwing interactions on aerodynamic forces and power in hovering dragonfly flight. Phys. Rev. Lett..

[17] Ellington C.P. (1999). The novel aerodynamics of insect flight: Applications to micro-air vehicles. J. Exp. Biol..

[18] Bode-Oke A.T., Zeyghami S., Dong H. (2017). Aerodynamics and flow features of a damselfly in takeoff flight. Bioinspiration Biomim..

[19] Bennet-Clark H.C., Daws A.G. (1999). Transduction of mechanical energy into sound energy in the cicada cyclochila australasiae. J. Exp. Biol..

[20] Williams K.S., Simon C. (1995). The Ecology, Behavior, and Evolution of Periodical Cicadas. Annu. Rev. Entomol..

[21] Bartholomew G.A., Barnhart M.C. (1984). Tracheal Gases, Respiratory Gas Exchange, Body Temperature and Flight in Some Tropical Cicadas. J. Exp. Biol..

[22] Kelleher S.M., Habimana O., Lawler J., O’ Reilly B., Daniels S., Casey E., Cowley A. (2016). Cicada Wing Surface Topography: An Investigation into the Bactericidal Properties of Nanostructural Features. ACS Appl. Mater. Interfaces.

[23] Sane S.P., Dieudonné A., Willis M.A., Daniel T.L. (2007). Antennal mechanosensors mediate flight control in moths. Science.

[24] Bode-Oke A.T., Dong H. (2020). The reverse flight of a monarch butterfly (*Danaus plexippus*) is characterized by a weight-supporting upstroke and postural changes. J. R. Soc. Interface.

[25] Bode-Oke A.T., Zeyghami S., Dong H. (2018). Flying in reverse: Kinematics and aerodynamics of a dragonfly in backward free flight. J. R. Soc. Interface.

[26] Schneider P. (1981). Flugmanöver der käfer. Mitteilungen Dtsch. Ges. Allg. Angew. Entomol..

[27] Tiegs O.W. (1955). The flight muscles of insects-their anatomy and histology; with some observations on the structure of striated muscle in general. Philos. Trans. R. Soc. London. Ser. B Biol. Sci..

[28] Zeyghami S., Babu N., Dong H. (2016). Cicada (*Tibicen linnei*) steers by force vectoring. Theor. Appl. Mech. Lett..

[29] Bode-Oke A., Dong H. (2018). On the Backward Flight of a Cicada: Kinematics and Aerodynamics. Bull. Am. Phys. Soc..

[30] Ma K.Y., Chirarattananon P., Fuller S.B., Wood R.J. (2013). Controlled Flight of a Biologically Inspired, Insect-Scale Robot. Science.

[31] Pfau H. (1991). Contributions of functional morphology to the phylogenetic systematics of Odonata. Adv. Odonatol..

[32] Wakeling J., Ellington C.P. (1997). Dragonfly flight. II. Velocities, accelerations and kinematics of flapping flight. J. Exp. Biol..

[33] Ros I.G., Bassman L.C., Badger M.A., Pierson A.N., Biewener A.A. (2011). Pigeons steer like helicopters and generate down- and upstroke lift during low speed turns. Proc. Natl. Acad. Sci. USA.

[34] Koehler C., Liang Z., Gaston Z., Wan H., Dong H. (2012). 3D reconstruction and analysis of wing deformation in free-flying dragonflies. J. Exp. Biol..

[35] Catmull E., Clark J. (1978). Recursively generated B-spline surfaces on arbitrary topological meshes. Comput.-Aided Des..

[36] Mittal R., Dong H., Bozkurttas M., Najjar F., Vargas A., von Loebbecke A. (2008). A versatile sharp interface immersed boundary method for incompressible flows with complex boundaries. J. Comput. Phys..

[37] Menzer A., Ren Y., Guo J., Tobalske B.W., Dong H. (2022). Wing Kinematics and Unsteady Aerodynamics of a Hummingbird Pure Yawing Maneuver. Biomimetics.

[38] Li C., Dong H., Zhao K. (2018). A balance between aerodynamic and olfactory performance during flight in Drosophila. Nat. Commun..

[39] Dong H., Mittal R., Najjar F.M. (2006). Wake topology and hydrodynamic performance of low-aspect-ratio flapping foil. J. Fluid Mech..

[40] Menzer A., Gong Y., Fish F.E., Dong H. (2022). Bio-inspired propulsion: Towards understanding the role of pectoral fin kinematics in manta-like swimming. Biomimetics.

[41] Hunt J.C., Wray A., Moin P. (1988). Eddies, streams, and convergence zones in turbulent flows. Cent. Turbul. Res. Rep..

[42] Brackenbury J. (1995). Insects in Flight.

[43] Willmott A.P., Ellington C.P. (1997). The mechanics of flight in the hawkmoth *Manduca sexta*. I. Kinematics of hovering and forward flight. J. Exp. Biol..

[44] Sane S.P., Dickinson M.H. (2001). The control of flight force by a flapping wing: Lift and drag production. J. Exp. Biol..

[45] Zhao L., Huang Q., Deng X., Sane S.P. (2010). Aerodynamic effects of flexibility in flapping wings. J. R. Soc. Interface.

[46] Hsu S.-J., Thakur N., Cheng B. (2019). Speed control and force-vectoring of bluebottle flies in a magnetically levitated flight mill. J. Exp. Biol..

[47] Grodnitsky D.L. (1995). Evolution and Classification of Insect Flight Kinematics. Evolution.

[48] Jantzen B., Eisner T. (2008). Hindwings are unnecessary for flight but essential for execution of normal evasive flight in Lepidoptera. Proc. Natl. Acad. Sci. USA.

[49] Moore T.E., Huber F., Weber T., Klein U., Back C. (1993). Interaction between visual and phonotactic orientation during flight in *Magicicada cassini* (*Homoptera*: *Cicadidae*). Great Lakes Entomol..

[50] Caetano J.V., de Visser C.C., Remes B.D., De Wagter C., Van Kampen E.-J., Mulder M. Controlled flight maneuvers of a Flapping Wing Micro Air Vehicle: A step towards the DelFly II Identification. Proceedings of the AIAA Atmospheric Flight Mechanics (AFM) Conference.

[51] Bomphrey R.J., Taylor G.K., Thomas A.L.R. (2009). Smoke visualization of free-flying bumblebees indicates independent leading-edge vortices on each wing pair. Exp. Fluids.

[52] Zeyghami S. (2015). Wing in the Loop: Integrating the Wing into Dynamics of Insect Flight. Ph.D. Thesis.

[53] Ellington C.P. (1984). The Aerodynamics of Flapping Animal Flight. Am. Zool..

[54] Ellington C.P. (1984). The aerodynamics of hovering insect flight. III. Kinematics. Philos. Trans. R. Soc. Lond. B Biol. Sci..

[55] Chen D., Kolomenskiy D., Nakata T., Liu H. (2017). Forewings match the formation of leading-edge vortices and dominate aerodynamic force production in revolving insect wings. Bioinspiration Biomim..

[56] Sato M., Azuma A. (1997). The flight performance of a damselfly *Ceriagrion melanurum* Selys. J. Exp. Biol..

[57] Russell D.B. (2004). Numerical and Experimental Investigations into the Aerodynamics of Dragonfly Flight. Ph.D. Thesis.

[58] Azuma A., Watanabe T. (1988). Flight Performance of a Dragonfly. J. Exp. Biol..

[59] Hefler C., Noda R., Shyy W., Qiu H. Unsteady Vortex Interactions for Performance Enhancement of a Free Flying Dragonfly. Proceedings of the ASME 2017 Fluids Engineering Division Summer Meeting.

[60] Meng X., Sun M. (2016). Wing Kinematics, Aerodynamic Forces and Vortex-wake Structures in Fruit-flies in Forward Flight. J. Bionic Eng..

[61] Willmott A.P., Ellington C.P., Thomas A.L. (1997). Flow visualization and unsteady aerodynamics in the flight of the hawkmoth, Manduca sexta. Philos. Trans. R. Soc. B Biol. Sci..

[62] Aono H., Liu H. (2006). Vortical Structure and Aerodynamics of Hawkmoth Hovering. J. Biomech. Sci. Eng..

[63] Young J., Walker S.M., Bomphrey R.J., Taylor G.K., Thomas A.L.R. (2009). Details of Insect Wing Design and Deformation Enhance Aerodynamic Function and Flight Efficiency. Science.

[64] Zheng L., Hedrick T.L., Mittal R. (2013). Time-Varying Wing-Twist Improves Aerodynamic Efficiency of Forward Flight in Butterflies. PLoS ONE.

