# Supplementary figures and images for: Postural Change of the Annual Cicada (Tibicen linnei) Helps Facilitate Backward Flight
Source: Biomimetics (Basel). 2024 Apr 14;9(4):233. doi: 10.3390/biomimetics9040233 (PMC11048523; doi:10.3390/biomimetics9040233)

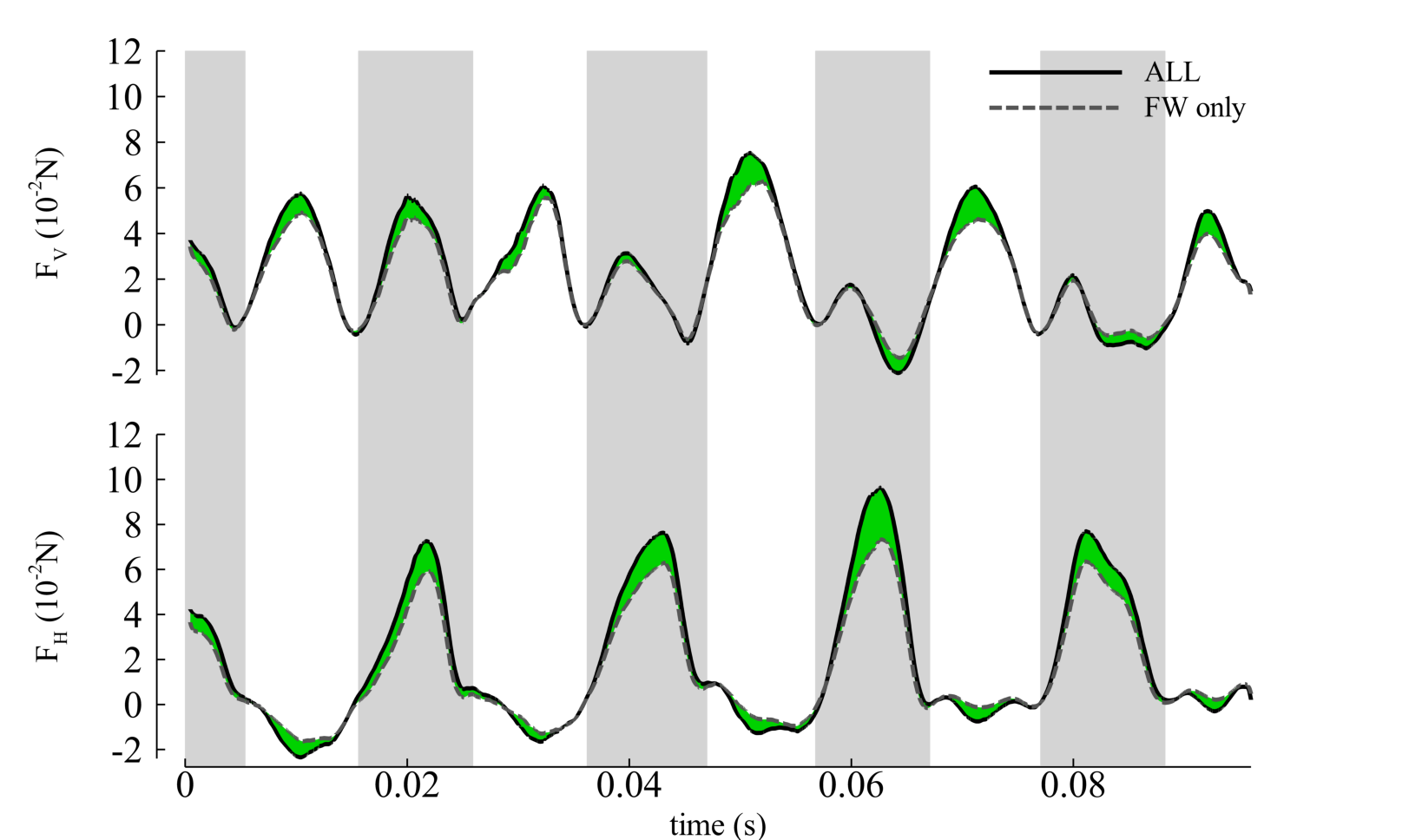

Supplement: Supplementary file 1 [file biomimetics-09-00233-s001.zip › ESM/FigureS1_CCD#2-Forewing versus hindwing force.png]
